# Supplementary material for: Intestinal Myo-Inositol Metabolism and Metabolic Effects of Myo-Inositol Utilizing Anaerostipes rhamnosivorans in Mice
Source: Int J Mol Sci. 2025 Sep 24;26(19):9340. doi: 10.3390/ijms26199340 (PMC12524588; doi:10.3390/ijms26199340)
Supplement: Supplementary file 1 [file ijms-26-09340-s001.zip › ijms-3865241-supplementary.pdf]

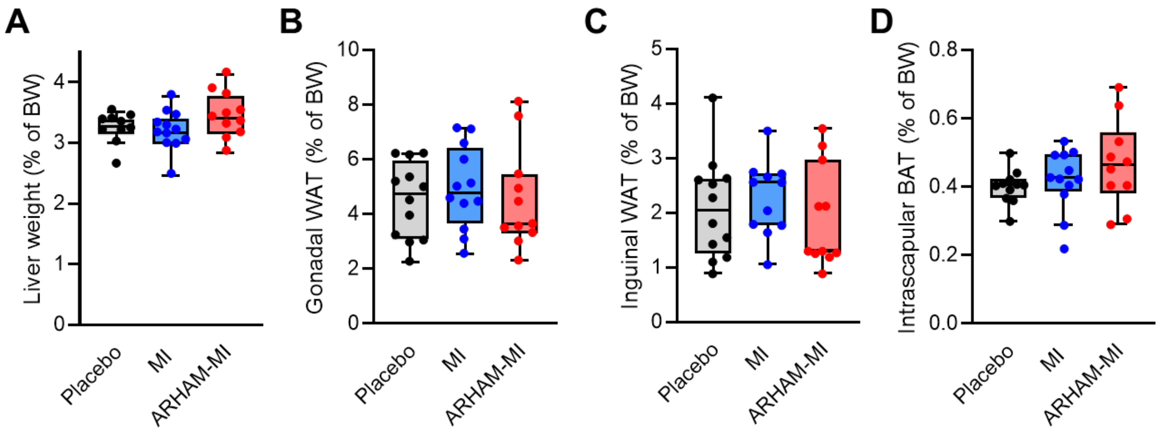

Figure S1: Effects of 13-week treatment with ARHAM-MI or MI alone on the relative weight of (A) liver, (B) gonadal white adipose tissue (WAT), (C) inguinal WAT, and (D) intrascapular brown adipose tissue (BAT) of HFD mice. Data shown as boxplots with 25th–75th percentiles and whiskers following Tukey's method; n = 10-12 mice per group; *, $p<0.05$ in 1-way ANOVA compared to ARHAM-MI; BW, body weight.

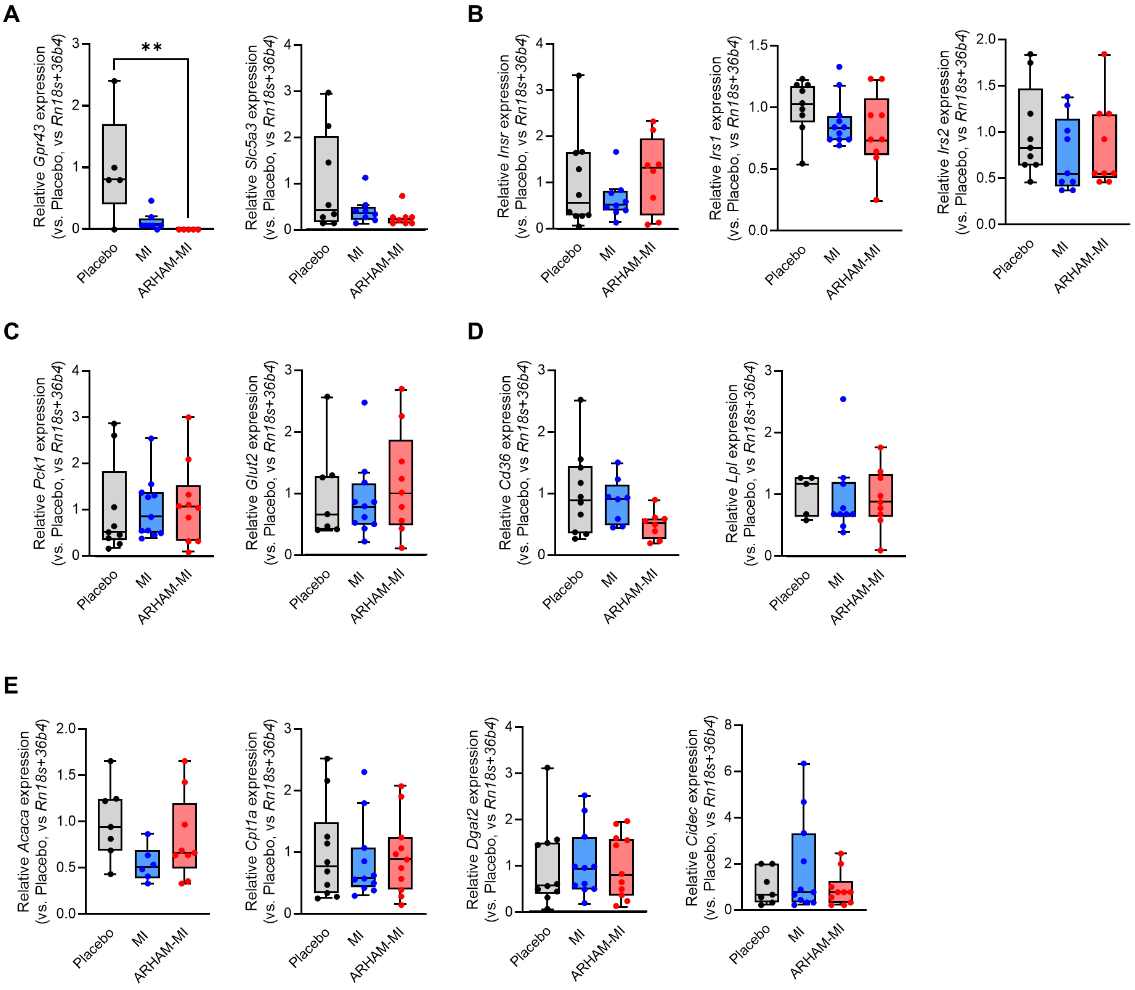

Figure S2: Differences in hepatic mRNA expression of the genes encoding the MI transporter Slc5a3 and the SCFA receptor Gpr43 (A), the insulin receptor (Insr) and its downstream substrates Irs1 and Irs2 (B), the gluconeogenic protein phosphoenolpyruvate carboxykinase 1 (Pck1) and the glucose transporter-2 (C), proteins involved in cellular lipid uptake (D), and the proteins involved in cellular lipid synthesis and oxidation (E) of HFD mice treated for 13 weeks with ARHAM-MI or MI alone. * $p < 0.05$ in 1-way ANOVA compared to ARHAM-MI. Data shown as averages ± SEM or boxplots with 25th–75th percentiles and whiskers following Tukey's method, n = 8-12 mice per group.

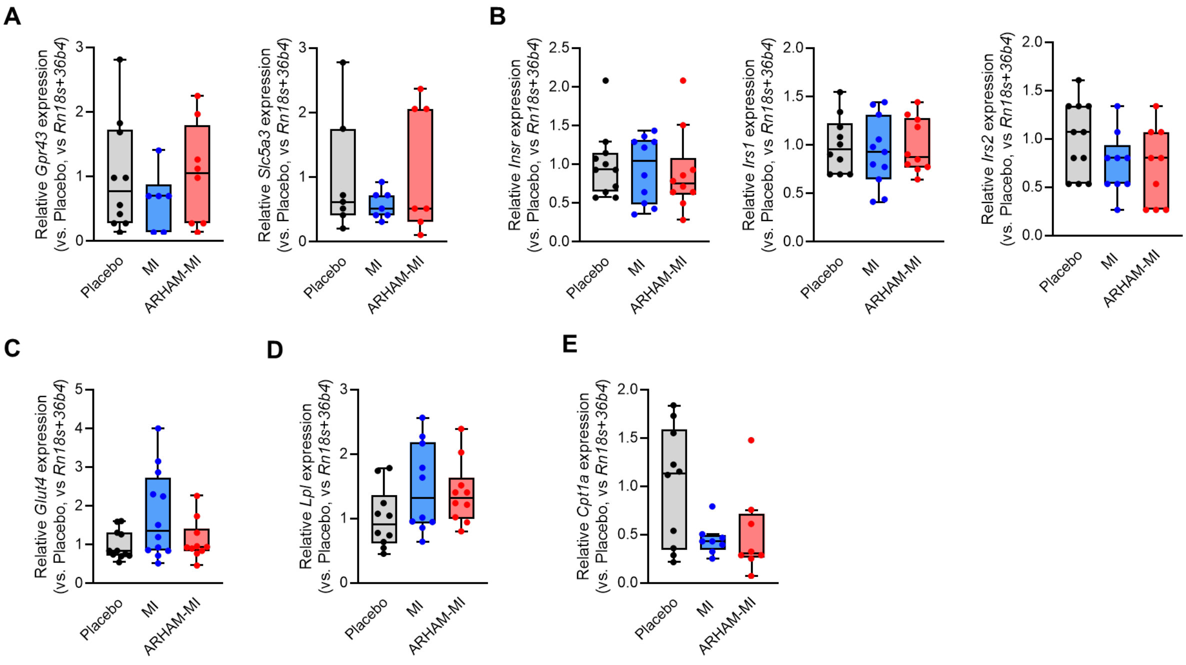

Figure S3: Differences in skeletal muscle mRNA expression of the genes encoding the MI transporter Slc5a3 and the SCFA receptor Gpr43 (A), the insulin receptor (Insr) and its downstream substrates Irs1 and Irs2 (B), the glucose transporter-4 (C), lipoprotein lipase (Lpl) (D), and the rate-controlling protein of fatty acid oxidation carnitine palmitoyltransferase-1A (Cpt1a) of HFD mice treated for 13 weeks with ARHAM-MI or MI alone. * $p < 0.05$ in 1-way ANOVA compared to ARHAM-MI. Data shown as averages ± SEM or boxplots with 25th–75th percentiles and whiskers following Tukey's method, n = 8-12 mice per group.

Table S1: Macronutrient compositions of the high fat diet (HFD).

Nutrient	
Proteins (%)	26.2
Carbohydrates (%)	25.6
Fat (%)	34.9
Fiber (%)	6.5

Table S2: Sequences of primers for ARHAM quantification

**Gene**	**Forward primer**	**Reverse primer**
16s	CGGTGAATACGTTCYCGG	GGWTACCTTGTTACGACTT
ARHAM	CTGCACTCTAGCATTACAGT	GCGTAGGTGGCATGATAAGT

Table S3: Sequences of primers for qPCRs of mouse tissue

Gene	Forward primer	Reverse primer
Acaca	GGGAGAAACAGGGAGGAAG	TCGAAAGTCACCCCGAATAG
Cd36	TGGCTAAATGAGACTGGGACC	GGCCATCTCTACCATGCCAA
Cidec	CCCATCAGAACAGCGCAAGAA	TCATAGAGGGTTGCCTTCACG
Cpt1a	AGAGGGGAGGACAGAGACTG	TCTGCTCTGCCGTTGTTGTG
Dgat2	ATCCTTCCTGGTGCTAGGAGT	GCCAGCCAGGTGAAGTAGAG
Glut2	AATGGTCGCCTCATTCTTTG	AGCCAACATTGCTTTGATCC
Glut4	GACGGACACTCCATCTGTTG	GCCACGATGGAGACATAGC
Gpr43	CCTTGATCCTCACGGCCTAC	AGTCCGCCAGGGTCAGATTA
Insr	TTGAGGTGGGAACCCTACTG	ATCCTGCCCATCAAACTCTG
Irs1	CTATGCCAGCATCAGCTTCC	TTGCTGAGGTCATTTAGGTCTTC
Irs2	ACAACCTATCGTGGCACCTC	CCATGAGACTTAGCCGCTTC
Lpl	CTCGCTCTCAGATGCCCTAC	CCACTGTGCCGTACAGAGAA
Pck1	ATGTGTGGGCGATGACATT	AACCCGTTTTCTGGGTTGAT
Pgc1a	TCAGAACCATGCAGCAAACC	AGGAGGGTCATCGTTTGTGG
Ppara	CTGAFACCCTCGGGGAAC	AAACGTCAGTTCACAGGGAAG
Rn18s	CACTTTTGGGGCCTTCGTG	GCAAAGGCCCAGAGACTCATT
Rplp0	GGACCCGAGAAGACCTCCTT	GCACATCACTCAGAATTTCAATGG
Slc5a3	GCCGTAGTGGCCCTGTATTT	CGGCCAGGAAGTATCCACTC

Acaca, acetyl-CoA carboxylase α; Cd36, cluster of differentiation 36; Cidec, cell death inducing DFFA like effector c; Cpt1a, carnitine palmitoyltransferase 1a; Dgat2, diacylglycerol O-acyltransferase 2; Glut, glucose transporter; Gpr43, G-protein coupled receptor 43; Insr, insulin receptor; Irs, insulin receptor substrate; Lpl, lipoprotein lipase; Pck1, phosphoenolpyruvate carboxykinase 1; Pgc1a, peroxisome proliferator-activated receptor gamma coactivator 1α; Ppara, peroxisome proliferator-activated receptor α; Rn18s, 18S ribosomal RNA; Rplp0, Ribosomal protein lateral stalk subunit P0 (36b4); Slc5a3, solute carrier 5a3.
